# Executive function training in very preterm children: a randomized controlled trial

**DOI:** 10.1007/s00787-020-01561-0

**Published:** 2020-05-26

**Authors:** Carolien A. van Houdt, Aleid G. van Wassenaer-Leemhuis, Jaap Oosterlaan, Marsh Königs, Corine Koopman-Esseboom, A. R. Céleste Laarman, Anton H. van Kaam, Cornelieke S. H. Aarnoudse-Moens

**Affiliations:** 1 Department of Neonatology, Emma Children’s Hospital, Amsterdam UMC, University of Amsterdam, Meibergdreef 9, Amsterdam, The Netherlands; 2 Emma Neuroscience Group, Emma Children’s Hospital, Amsterdam UMC, University of Amsterdam, Meibergdreef 9, Amsterdam, The Netherlands; 3 Department of Pediatrics, Amsterdam UMC, University of Amsterdam, Emma Neuroscience Group at Emma Children’s Hospital, Amsterdam Reproduction and Development, Meibergdreef 9, Amsterdam, The Netherlands; 4 Clinical Neuropsychology Section, Vrije Universiteit Amsterdam, Van der Boechorststraat 7, Amsterdam, The Netherlands; 5 Department of Neonatology, University Medical Center Utrecht, Heidelberglaan 100, Utrecht, The Netherlands; 6 Department of Neonatology, Emma Children’s Hospital, Amsterdam UMC, Vrije Universiteit Amsterdam, de Boelelaan 1117, Amsterdam, The Netherlands; 7 Psychosocial Department, Emma Children’s Hospital, Amsterdam UMC, University of Amsterdam, Meibergdreef 9, Amsterdam, The Netherlands

**Keywords:** Intervention, Premature, Attention, Behavior, Arithmetic, Reading

## Abstract

Objective of the current study was to assess whether game-formatted executive function (EF) training, is effective in improving attention, EF and academic performance in very preterm and/or extremely low birthweight children aged 8–12 years. A multi-center, double-blind, placebo- and waitlist controlled randomized trial (NTR5365) in two academic hospitals in The Netherlands was performed. Eighty-five very preterm children with parent-rated attention problems on the Child Behavior Checklist were randomized to one of three treatment conditions: EF training, placebo training or waitlist condition. EF or placebo training was completed at home (6 weeks, 25 sessions of 30–45 min each). At baseline, 2 weeks after training or being on the waitlist, and five months after first follow-up visit, children underwent assessments of primary outcomes (parent and teacher ratings of attention) and secondary outcomes (parent and teacher ratings of daily-life EF, computerized EF tasks and academic performance). Linear mixed model analyses were performed for all outcome measures. There were no significant differences in improvement over time on parent- and teacher ratings of attention, parent- and teacher ratings of daily-life EF, computerized EF tasks, and academic performance (arithmetic and reading) between the EF training, placebo training and waitlist condition. In conclusion, game-formatted EF training does not improve attention, EF or academic performance in very preterm children with parent-rated attention problems.

**Electronic supplementary material:**

The online version of this article (10.1007/s00787-020-01561-0) contains supplementary material, which is available to authorized users.

## Introduction

In Western countries, 0.7–1.4% of all live born children are born very preterm (gestational age (GA) < 32 weeks) [1]. Due to advances in medical care, survival rates have increased and approximately 65% of children born extremely preterm do not develop major disabilities [2]. However, more subtle problems in various domains are frequently encountered [3], which may have a significant negative impact on children’s and parents’ lives. One of the main and most persistent problems very preterm children encounter are problems in attentional functioning [4, 5]. Very preterm children have a two to four times higher risk of a diagnosis of Attention Deficit/Hyperactivity Disorder (ADHD) [6, 7], particularly the inattentive subtype [8], and an increased risk for an Autism Spectrum Disorder (ASD) diagnosis [8, 9]. Also in ASD, attentional problems are common [10] and several studies have shown high comorbidity rates of ADHD in children with ASD [11–15]. Although very preterm children’s behavioral attentional symptoms may resemble those of children with ADHD or ASD for some aspects, such as inattentive symptoms, the biological underpinnings seem to differ. For example, a recent study, comparing adolescents with ADHD and adolescents born preterm on electrophysiological measures associated with attentional and inhibitory processing, found that preterm adolescents showed both impairments overlapping with those found in adolescents with ADHD and impairments unique for the preterm adolescents [16]. Moreover, the clinical presentation is also different. For example, the association between oppositional defiant disorder (ODD) and conduct disorder (CD) is much stronger in the general population than in children born extremely preterm [8]. Thus, interventions that were ineffective in children with ADHD may be effective in very preterm children with attention problems and vice versa.

Deficits in executive functions (EFs) are considered to play an important role in the complex neuropsychology of both ADHD and ASD [17–26]. Executive functioning is an umbrella term for a set of higher-order cognitive functions that allow for top-down, goal-directed behavior, with core functions including working memory, inhibition and cognitive flexibility [27]. Research has indicated that poor EFs are strongly associated with the attentional problems of very preterm children as well [28–30]. Both ASD and ADHD are neurodevelopmental disorders that affect key fronto-striatal and fronto-parietal circuits that are important for EFs [31], and very preterm birth has been shown to affect white matter network integrity and brain structures associated with EFs [32–39].

Computerized training interventions to improve EFs may therefore be one way to address the attentional problems very preterm children encounter. Cogmed Working Memory Training (CWMT) is widely used in children with Attention Deficit/Hyperactivity Disorder (ADHD) [40]. It has been coined as a promising computerized EF training for attentional problems [41], although more recent studies suggest that it might not be as effective as previously thought [42]. In very preterm children, a recent randomized controlled trial showed no effects of CWMT on any outcome measure, including attentional functioning [43]. However, CWMT solely trains working memory, while very preterm children show problems in inhibition and cognitive flexibility as well [5, 44, 45]. Recently, the BrainGame Brian training was developed [46], targeting not only working memory, but also inhibition and cognitive flexibility. BrainGame Brian training further expands upon the CWMT by adding game elements and using strong and immediate reinforcements to optimize children’s motivational state. To date, effects of BrainGame Brian training have been assessed in three studies including children with clinically diagnosed ADHD and Autism Spectrum Disorder (ASD). These studies have consistently shown improvements in visuospatial working memory, although without consistent effects on other EFs or other untrained functions [47–49]. Previously, our group showed in a small sized non-randomized pilot study that BrainGame Brian training was a feasible intervention for very preterm children. Clinically significant benefits for visuospatial working memory were found [50].

The current study examined, using a double-blind, randomized controlled design including both a placebo- and a waitlist condition, whether game-formatted EF training (i.e., BrainGame Brian training) improves attentional functioning in very preterm (GA < 30 weeks) and/or extremely low birthweight children (birthweight < 1000 g) aged 8 to 12 years. We also examined the effects of game-formatted EF training on secondary outcome measures. Because EF training is believed to improve attentional functioning through improving EFs, we examined whether game-formatted EF training indeed improved EFs. Last, as both attentional problems and EF deficits are strongly linked to worse academic performance in very preterm children [28, 29, 51–54], we also investigated whether game-formatted EF training improves academic performance, more specifically arithmetic and reading performance.

## Methods

### Trial design and ethical considerations

This multi-center, double-blind, placebo and waitlist controlled randomized trial was conducted in two academic hospitals in The Netherlands (Amsterdam University Medical Centers and University Medical Center Utrecht). Medical Ethical Committees in both centers approved the study protocol and execution of the study procedures was according to the Declaration of Helsinki. The trial was registered in the Dutch Trial Registry (NTR5365). CONSORT guidelines were followed.

### Participants

Parents of 7–12 year old children born very preterm and/or with extremely low birthweight (in short: very preterm children) who were admitted to the Neonatal Intensive Care Unit (NICU) in one of the two participating centers and who joined the neonatal follow-up program, were asked to complete the Dutch version of the Child Behavior Checklist (CBCL6-18 [55]). Children with parent-rated attention problems on the CBCL6-18 (*T* ≥ 55 on Attention Problems scale [56], as research has suggested this T-score is the optimal cut-off score for ADHD screening [56]) were eligible for this study as soon as they reached a minimum chronological age of 8 years. Exclusion criteria were estimated Intelligence Quotient (IQ) < 80, motor problems too profound to allow use of a computer and no Dutch language use in the home situation.

### Randomization and blinding

Children meeting inclusion criteria were randomly assigned to one of three treatment conditions: training-, placebo- or waitlist condition. Allocation to treatment conditions was stratified by age (below or above 10.5 years of age) and severity of attention problems (Attention Problems T-score below or above 65), with equal proportions of children allocated to each condition within each stratum. To ensure blinding to training- or placebo condition, parents were only informed about whether their child was randomized to either one of two training conditions or the waitlist condition and in case more children from the same family were included in the study, one of those was randomized and the other was allocated to the same condition. A random number generator was used to generate randomization lists. A researcher not otherwise involved in this study was responsible for randomization and handed the test assistant a sealed envelope with a note stating ‘waitlist’ or a login and password, which was opened by the child and parents after baseline assessment. All staff was blinded to training or placebo assignment, including the person involved in randomization. Test assistants that played the first training session with the child were deblinded because of differences in training tasks (see below) between training- and placebo condition and were not involved in follow-up assessments of these children. Parents, children and test administrators were aware of a child’s allocation to the waitlist condition. Data were analyzed blinded to treatment allocations.

## Intervention

### BrainGame Brian training

Braingame Brian is a game-formatted computerized EF training, performed by the child at home. An elaborate description of the BrainGame Brian training can be found in Prins et al. [46]. BrainGame Brian is a game-world environment, in which three EF training tasks, one for each core EF, are embedded. In each of 25 training sessions, children have to help the main character, Brian, to solve problems for other game-world characters. To do so, they play the three EF training tasks, which leads to the creation of an invention that solves the problem. For example, Brian meets a character that has a problem shearing his sheep. By playing the three EF training tasks, a sheep-shearing machine is invented, which solves the problem and remains visible in the game-world throughout the rest of the sessions to optimize motivational state and self-control of the children. In each session, children play the three training tasks twice. In total, 25 sessions are played, and the child and parents were instructed to play the BrainGame Brian training 4 times a week (approximately 30–45 min per session). After each session, session data were saved to a database, which was accessible for researchers to monitor fidelity to the training regimen. There was no additional support of researchers regarding motivation and/or feedback out of the computer game interface. For our randomized controlled trial, two versions of the BrainGame Brian EF training were used: the BrainGame Brian EF training, and a placebo version of the BrainGame Brian EF training. In the placebo version of the BrainGame Brian EF training, everything is identical to the BrainGame Brain EF training, except that the EF training tasks were replaced by placebo versions of these tasks. See below for the descriptions of the training tasks for both versions of the training.

### Training tasks in EF training condition

In the working memory task, children are asked to repeat a sequence of dots on a 4 × 4 grid in a specific way (e.g., forwards, backwards). The instructions for this task change every five sessions to increase working memory demands. In the inhibition task, children are asked to press a button in a specific time window (target), but to refrain from pressing that button when a stop signal is presented. In the cognitive flexibility task, children are asked to sort objects according to one of two rules, with the sorting rule changing every three to five trials. After each block of all three training tasks, difficulty level of each task is automatically adjusted to the child’s performance.

### Training tasks in placebo condition

In the placebo versions of the EF training tasks, the elements actually training the EFs were removed, and difficulty level was set at the lowest difficulty level for all sessions regardless of the child’s performance. In the placebo version of the working memory task, children are asked to repeat sequences in the same order as presented (which requires short-term memory, not working memory). This instruction remains the same throughout all training sessions. In the placebo version of the inhibition task, no stop signals are presented (thus children do not have to inhibit responses). In the placebo version of the cognitive flexibility task, the sorting rule never changes (thus no cognitive flexibility is required).

### Waitlist condition

Children in the waitlist condition do not play the BrainGame Brian training and were instructed to perform the same activities in the waiting period as they normally do.

### Procedure

After written informed consent was obtained from parents and, if applicable, children aged 12 years, and verbal informed consent was obtained from children below 12 years of age, participants completed a baseline neurocognitive assessment including measures of IQ, EF and academic functioning. Parents and teachers were asked to fill out questionnaires on children’s attention and daily-life EF behavior. If children were randomized to the training- or placebo condition, a house visit was made to instruct children and parents and play the first session. In two follow-up visits, the neurocognitive assessment was repeated except for IQ, which was only administered at baseline. The first follow-up assessment (T1) was approximately two weeks after the last training session (approximately 2 months after baseline assessment for children in the waitlist condition) and the second follow-up assessment (T2) was approximately 5 months after the first follow-up assessment.

### Measures

Primary outcome was parent- and teacher-rated attention as measured by the Strengths and Weaknesses of ADHD-symptoms and Normal Behavior (SWAN) questionnaire [57]. Secondary outcomes were (1) daily-life EF reported by parents and teachers as measured with the Behavior Rating Inventory of Executive Function (BRIEF) [58], (2) verbal working memory as measured using the Digit Span Backward subtest of the WISC-III-NL [59], (3) visuospatial working memory as measured using the Grid Task, backwards condition [60], (4) inhibition as measured with the Stop Signal Task [61], (5) cognitive flexibility as measured with the Multisensory Integration Test (MSIT) [62], (6) arithmetic as measured with the TempoTest Automatiseren (TTA) [63] and (7) Technical reading as measured with the Brus Een Minuut Test (B-EMT) [64]. Detailed descriptions of tasks and outcome measures can be found in Supplementary Material 1. Other secondary outcomes related to untrained functions were assessed as well, but were not included in the current paper on main outcome measures [65].

### IQ, demographic characteristics and medical characteristics of neonatal period

IQ was estimated with a short-form of the Dutch Wechsler Intelligence Scale for Children, third edition (WISC-III-NL [59]), comprising the subtests Vocabulary and Block Design. Parents provided information on demographics. The Digit Span [59] and Grid Task [60] forwards conditions (repeating a sequence of spoken digits or dots on a grid in forwards order, respectively), were administered to provide maximal forwards span length. Neonatal medical data were obtained from medical records.

### Statistical analyses

Sample size calculations determined that, to detect a medium-sized intervention effect (Cohen’s *d* = 0.5) on our primary outcome measure with a within-subject correlation of 0.295 [50] with a power of 80% and a significance level of 0.05, 39 children in each intervention arm were needed.

IBM SPSS Statistics version 24 was used for the statistical analyses [66]. Outliers were winsorized at three SDs [67]. Data were missing for less than 5% of children, except for: the baseline assessments of the teacher SWAN (12.9%), teacher BRIEF (14.1%) and Stop Signal Task (9.4%); the first follow-up assessments of the teacher SWAN (30.1%), teacher BRIEF (30.1%), Grid Task Backwards (6.8%) and MSIT (9.6%); the second follow-up assessments of the teacher SWAN (44.9%), parent BRIEF (5.8%), teacher BRIEF (40.6%), Grid Task Backwards (17.4%), Stop Signal Task (10.1%) and MSIT (7.2%). Missing data were not imputed.

Data were analyzed on intention-to-treat basis. To assess whether attrition from the study was selective, children that did and did not complete all assessments were compared on all demographic and neonatal medical characteristics with independent *t* tests and chi-square tests.

To assess whether BrainGame Brian training improves attention, EF and academic performance, linear mixed model analyses were run for all primary and secondary outcome measures with a random intercept to account for dependency in the data due to family bonds, and fixed factors for treatment condition (training, placebo or waitlist condition), time (baseline, first follow-up and second follow-up assessment) and the interaction between the two. All available data (also of participants with missing data) were used in the linear mixed model analyses. Estimated marginal means were reported to aid interpretation of the results.

## Results

### Participants

**Fig. 1:**
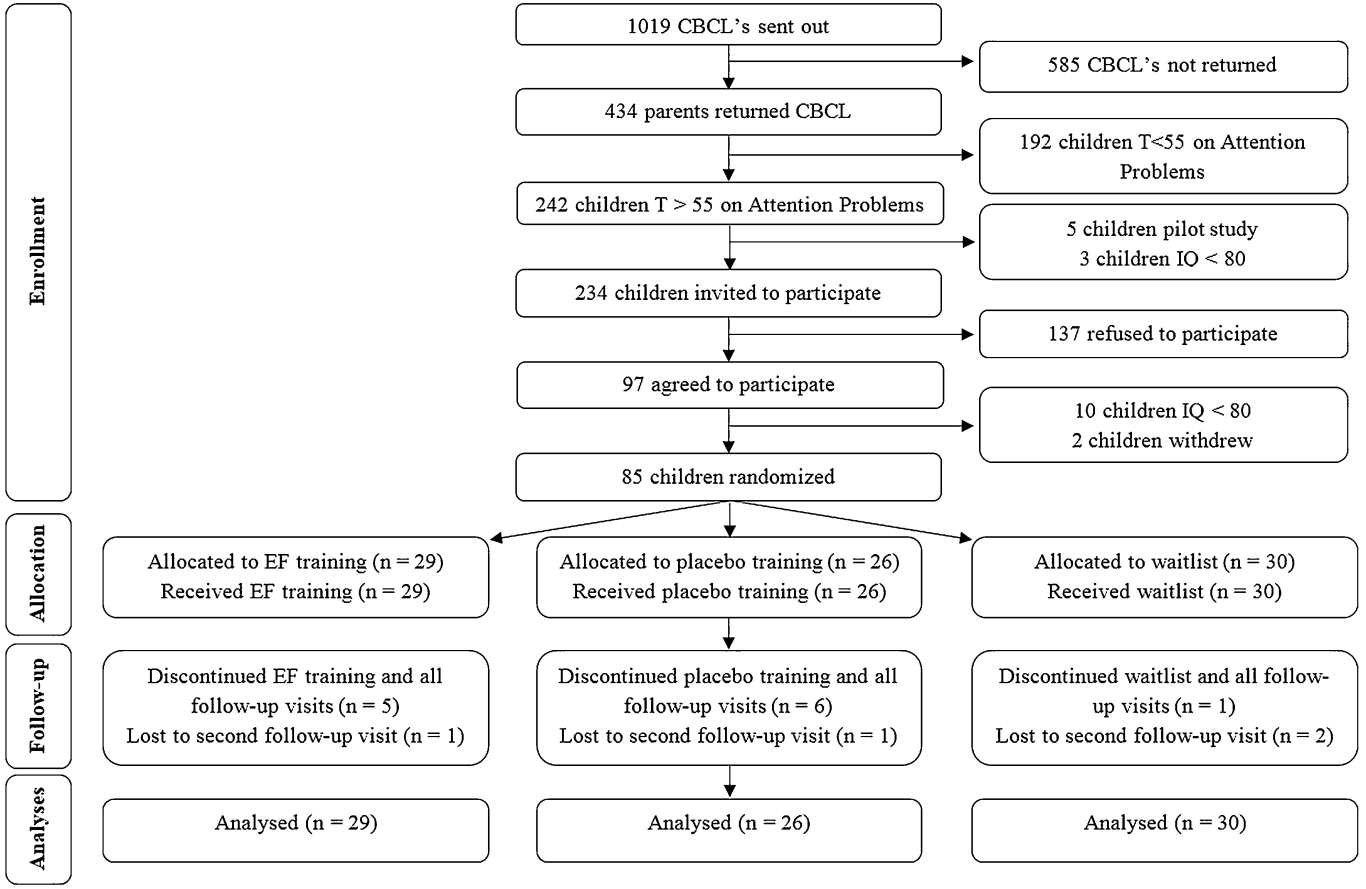
CONSORT flow diagram. *CBCL *child behavior checklist, *IQ* intelligence quotient, *EF* Executive function. Re-used from Van Houdt et al. [65]. Copyright 2019 by van Houdt, Aarnoudse-Moens, van Wassenaer-Leemhuis, Laarman, Koopman-Esseboom, van Kaam and Oosterlaan

Participants’ flow through the study process is depicted in Fig. 1. In short, 434 out of 1,019 parents returned the completed questionnaire. Main reasons not to return the questionnaire were no time or no interest. Of 234 children with elevated attention scores that were invited to participate, 97 agreed. Reasons to not participate were: already busy schedules for the child and/or family, or no interest. Two children withdrew from the study before randomization. Ten children were excluded due to estimated IQ < 80. The remaining 85 children were randomized to a treatment condition (see Fig. 1). All children in the EF training and placebo training conditions that had at least one follow-up assessment, completed all 25 training sessions.

Randomization allocated 29 children to the training condition, 26 children to the placebo condition and 30 children to the waitlist condition. Twelve children withdrew from the study before the first follow-up visit and four withdrew before the second follow-up visit. Reasons for withdrawal were: not able to incorporate training sessions into busy schedule or child not wanting to complete training (*n* = 9), no time or willingness to schedule follow-up visit(s) at appropriate time-point(s) (*n* = 5) or severe illness discovered (*n* = 2). Of the included children 81% completed all assessments. Attrition analyses showed no differences in demographic and neonatal medical characteristics between children that did and did not complete all assessments (all *p* values > 0.14). As no data from nonparticipating children could be assessed, we could not perform attrition analyses regarding the representativeness of our sample. However, when compared to very preterm children included in previous cohort-studies of our research group, the GA and BW of the included children was similar (GA around 28 weeks and BW around 1000 g [3, 68]). Parental education level of our sample was high in 61% of children, compared to approximately 45% of the general Dutch population between 25–45 years of age being highly educated [69]. Assessments took place between October 2015 and September 2018. Demographics and neonatal medical characteristics are presented in Table 1.

**Table 1:** Demographic and neonatal medical characteristics for the three treatment groups

Measure	BGB-Training (*n* = 29)	BGB-Placebo (*n* = 26)	Waitlist (*n* = 30)
Demographic characteristics:			
GA (M, SD)	28.2 (1.3)	28.0 (1.0)	27.8 (1.4)
BW (gram; M, SD)	1026 (256)	1039 (179)	1049 (267)
Age (years; M, SD)	10.2 (1.2)	10.2 (1.3)	10.3 (1.1)
IQ (M, SD)	99.0 (13.6)	96.4 (11.7)	100.8 (11.1)
CBCL attention *T* score (M, SD)	62.8 (6.9)	64.0 (7.6)	64.4 (7.0)
Digit span forwards span length	5.1 (0.9)	5.1 (1.1)	5.1 (0.8)
Grid task forwards span length	4.2 (1.2)	4.0 (1.3)	4.6 (1.2)
Boys (*n*, %)	13 (45%)	16 (62%)	20 (67%)
Parental education level (*n*/total *n*)			
Low	6/29	4/25	1/28
Middle	3/29	5/25	11/28
High	20/29	16/25	16/28
Neonatal medical characteristics			
SGA (*n*, %)	8 (28%)	4 (15%)	4 (13%)
Ventilator support (*n*, %)	20 (69%)	17 (65%)	23 (77%)
BPD at 36 weeks PMA (*n*, %)	6 (21%)	4 (15%)	5 (17%)
IVH I or II	9 (31%)	6 (23%)	8 (27%)
IVH III or IV	0 (0%)	2 (8%)	1 (3%)
PVL I	1 (3%)	2 (8%)	0 (0%)
PVL II, III or IV	0 (0%)	0 (0%)	0 (0%)
Open ductus botalli treated	3 (10%)	12 (46%)	13 (43%)
Sepsis	17 (59%)	16 (62%)	20 (67%)

*BGB *BrainGame Brian, *GA* gestational age, *BW* birth weight, *IQ* intelligence quotient, *CBCL* child behavior check list, *SGA* small for gestational age, *BPD* broncho pulmonary dysplasia, *PMA* post menstrual age, *IVH* intra ventricular hemorrhage, *PVL* peri ventricular leukomalacia, *M* mean, *SD* standard deviation, *n* number

### Primary and secondary outcome measures

There were no significant differences over time between the three treatment conditions for both parent and teacher SWAN questionnaires, indicating no beneficial effects of the BrainGame Brian training as compared to the placebo or waitlist condition.

There were also no significant differences over time between the three treatment conditions for any of the EF measures, indicating no beneficial effects of the BrainGame Brian training as compared to the placebo or waitlist condition. Last, there were no significant differences over time between the three treatment conditions for both the TTA and B-EMT, indicating no beneficial effects of the BrainGame Brian training as compared to the placebo or waitlist condition.

There were significant main effects of time for teacher BRIEF Working Memory and Total Score, Grid Task Backwards, Stop Signal Task, TTA and B-EMT, all indicating better performance over time. There were significant main effects of group for the Grid Task Backwards and MSIT, both indicating poorer performance in the placebo training group than in the BrainGame Brian training group and the waitlist group, with no difference in performance between the latter two (see Tables 2 and 3).

**Table 2:** Baseline and follow-up data on the SWAN for the three intervention groups

Outcome measure	*T*0	*T*1	*T*2	*p* value effect of intervention condition	*p* value effect of time	*p* value interaction effect of intervention condition x time
SWAN parent						
Attention deficit (M, SE)				.17	.18	.91
BGB-Training	40.1 (1.4)	39.9 (1.5)	38.9 (1.5)			
BGB-Placebo	42.2 (1.4)	43.1 (1.5)	42.0 (1.6)			
Waitlist	42.6 (1.3)	43.8 (1.4)	42.0 (1.4)			
Hyperactivity/impulsivity (M, SE)				.69	.12	.69
BGB-Training	38.1 (1.5)	38.7 (1.6)	36.6 (1.6)			
BGB-Placebo	40.6 (1.6)	38.9 (1.7)	38.7 (1.7)			
Waitlist	39.7 (1.4)	39.2 (1.5)	38.8 (1.5)			
Total score (M, SE)				.29	.06	.98
BGB-Training	78.4 (2.5)	79.3 (2.6)	75.5 (2.6)			
BGB-Placebo	82.9 (2.5)	82.9 (2.7)	80.7 (2.8)			
Waitlist	82.3 (2.3)	83.0 (2.4)	80.7 (2.4)			
SWAN teacher						
Attention deficit (M, SE)				.17	.05	.58
BGB-Training	37.7 (1.6)	37.7 (1.6)	37.0 (1.8)			
BGB-Placebo	40.6 (1.7)	40.6 (1.9)	39.0 (2.1)			
Waitlist	42.3 (1.5)	42.8 (1.6)	39.0 (1.7)			
Hyperactivity/impulsivity (M, SE)				.35	.81	.41
BGB-Training	34.1 (1.9)	32.2 (2.0)	33.9 (2.2)			
BGB-Placebo	36.4 (2.0)	34.6 (2.4)	36.3 (2.7)			
Waitlist	36.7 (1.8)	38.3 (2.0)	35.4 (2.1)			
Total score (M, SE)				.18	.42	.38
BGB-Training	71.8 (3.0)	70.0 (3.2)	70.9 (3.5)			
BGB-Placebo	76.9 (3.2)	75.2 (3.7)	75.5 (4.2)			
Waitlist	78.9 (2.9)	81.1 (3.1)	74.3 (3.3)			

*SWAN* strengths and weaknesses of ADHD-symptoms and normal behavior, *BGB* BrainGame Brian, *M* mean, *SE* standard error, *T0* Time-point 0, i.e. baseline, *T1* time-point 1, i.e. first follow-up visit, *T2* time-point 2, i.e. second follow-up visit. See Fig. 1 for number of participants in each group at each time-point

**Table 3:** Baseline and follow-up data on EF and academic performance for the three intervention groups

Outcome measure	*T*0	*T*1	*T*2	*p* value effect of intervention condition	*p* value effect of time	*p* value interaction effect of intervention condition x time
BRIEF parent working memory (M, SE)				.33	.06	.61
BGB-Training	57.45 (1.68)	56.09 (1.74)	54.12 (1.83)			
BGB-Placebo	56.56 (1.70)	57.47 (1.87)	54.79 (1.90)			
Waitlist	58.75 (1.58)	59.13 (1.60)	58.28 (1.62)			
BRIEF parent inhibit (M, SE)				.20	.27	.74
BGB-Training	51.23 (2.05)	49.70 (2.12)	48.65 (2.20)			
BGB-Placebo	54.98 (2.07)	52.93 (2.24)	53.94 (2.27)			
Waitlist	54.27 (1.92)	54.57 (1.95)	53.55 (1.96)			
BRIEF parent cognitive flexibility (M, SE)				.57	.27	.64
BGB-Training	52.06 (2.00)	50.26 (2.08)	50.30 (2.17)			
BGB-Placebo	48.77 (2.02)	47.45 (2.22)	48.55 (2.25)			
Waitlist	51.34 (1.87)	51.08 (1.91)	48.56 (1.92)			
BRIEF parent total (M, SE)				.33	.54	.53
BGB-Training	52.41 (1.88)	51.57 (1.95)	49.75 (2.03)			
BGB-Placebo	52.75 (1.90)	53.48 (2.07)	53.99 (2.10)			
Waitlist	54.74 (1.77)	55.57 (1.79)	53.95 (1.81)			
BRIEF teacher working memory (M, SE)				.63	.001*	.96
BGB-Training	58.40 (3.03)	58.62 (3.20)	53.70 (3.27)			
BGB-Placebo	61.93 (3.30)	63.79 (3.55)	57.18 (3.94)			
Waitlist	60.00 (2.92)	59.37 (3.05)	54.42 (3.13)			
BRIEF teacher inhibit (M, SE)				.59	.67	.39
BGB-Training	49.43 (1.94)	48.79 (2.14)	51.02 (2.21)			
BGB-Placebo	53.08 (2.15)	52.00 (2.43)	51.03 (2.85)			
Waitlist	52.92 (1.88)	52.95 (2.03)	50.07 (2.12)			
BRIEF teacher cognitive flexibility (M, SE)				.83	.67	.96
BGB-Training	55.29 (2.77)	54.69 (3.08)	53.73 (3.19)			
BGB-Placebo	52.66 (3.08)	53.19 (3.49)	52.39 (4.12)			
Waitlist	52.51 (2.69)	54.38 (2.91)	50.90 (3.05)			
BRIEF teacher total (M, SE)				.59	.03*	.27
BGB-Training	52.60 (2.25)	53.15 (2.40)	51.72 (2.45)			
BGB-Placebo	54.84 (2.46)	57.95 (2.67)	54.52 (3.00)			
Waitlist	55.81 (2.17)	55.60 (2.28)	50.65 (2.35)			
Digit span backwards (M, SE)				.67	.91	.94
BGB-Training	16.19 (1.75)	16.77 (1.88)	17.77 (1.91)			
BGB-Placebo	17.67 (1.81)	16.50 (2.03)	17.04 (2.12)			
Waitlist	15.79 (1.68)	15.31 (1.71)	15.31 (1.78)			
Grid task backwards (M, SE)				.02*	.02*	.33
BGB-Training	35.30 (5.40)	50.88 (6.26)	56.76 (6.94)			
BGB-Placebo	27.17 (5.54)	30.98 (6.39)	34.57 (6.96)			
Waitlist	38.39 (5.15)	48.24 (5.23)	40.10 (5.60)			
Stop task						
SSRT (M, SE)				.99	.002*	.49
BGB-Training	323.4 (30.8)	293.3 (31.9)	288.9 (32.2)			
BGB-Placebo	312.4 (31.4)	302.0 (34.6)	270.3 (33.5)			
Waitlist	310.3 (30.0)	316.3 (29.6)	263.6 (30.2)			
# errors (M, SE)				.61	.01*	.32
BGB-Training	5.21 (1.26)	3.08 (1.43)	3.93 (1.40)			
BGB-Placebo	6.06 (1.30)	3.41 (1.69)	3.41 (1.50)			
Waitlist	6.66 (1.21)	6.30 (1.19)	3.35 (1.27)			
MSIT shifting accuracy loss (M, SE)				.002*	.25	.35
BGB-Training	3.4% (2.1%)	3.2% (2.3%)	3.1% (2.4%)			
BGB-Placebo	14.4% (2.2%)	12.9% (2.7%)	7.4% (2.5%)			
Waitlist	3.9% (2.0%)	5.5% (2.0%)	4.1% (2.1%)			
TTA (M, SE)				.95	< . 001*	.95
BGB-Training	90.3 (9.5)	97.6 (9.6)	101.3 (9.7)			
BGB-Placebo	93.0 (9.7)	103.1 (10.0)	105.9 (10.0)			
Waitlist	89.8 (9.0)	97.7 (9.0)	104.7 (9.1)			
B-EMT (M, SE)				.99	.001*	.99
BGB-Training	59.6 (3.7)	61.5 (3.8)	64.5 (3.8)			
BGB-Placebo	60.8 (3.7)	61.3 (3.9)	65.7 (4.0)			
Waitlist	60.1 (3.5)	61.7 (3.5)	65.3 (3.5)			

*BGB* BrainGame Brian, *BRIEF* Behavior Rating Inventory of Executive Function, *SSRT* stop signal reaction time, *MSIT* multisensory integration task, *B-EMT* Brus-Een minuut test, *TTA* tempo test automatiseren, *M* mean, *SE* standard error, *T0* time-point 0, i.e. baseline, *T1* time-point 1, i.e. first follow-up visit, *T2* time-point 2, i.e. second follow-up visit. See Fig. 1 for number of participants in each group at each time-point

*Significant at alpha < .05

## Discussion

The present study investigated whether game-formatted EF training improves attentional functioning in children born very preterm and/or with extremely low birthweight (in short: very preterm) with parent-rated attention problems. Additional outcomes included computerized EF tasks, parent- and teacher reported daily-life EF and academic performance. Results of our double-blind RCT provided no evidence for beneficial effects on any of the outcome measures assessed.

In children with ADHD, positive effects of both BrainGame Brian training and CWMT on working memory performance have been found [48, 70–72]. Because behavioral symptoms of inattention in children born very preterm resemble those of children with ADHD, we expected positive effects in children born very preterm. However, results of our study indicated no positive effects of the BrainGame Brian training in children born very preterm. Results of our study are in line with a randomized controlled trial investigating effects of CWMT in very preterm children, with no positive effects of CWMT on attentional functioning, working memory performance and academic performance [43]. Also, we recently analyzed our other secondary outcome measures including behavioral and emotional functioning and self-perceived competence [65]. None of these untrained functions were improved by BrainGame Brian training either. Our results are also in line with previous meta-analyses investigating the effect of CWMT on far transfer measures in children with ADHD [41, 42, 73, 74]. Despite the behavioral parallels between ADHD and symptoms of inattention in children born very preterm, the neurobiological underpinnings of these symptoms may actually be different. A recent study, for instance, using electroencephalography (EEG) measures associated with attentional and inhibitory processing, demonstrated that adolescents born preterm show unique patterns of aberrant neural activity as compared to adolescents with ADHD, suggestive of more generalized impairments in adolescents born preterm as compared to adolescents with ADHD [16]. This implies that interventions that are not effective in children with ADHD may be effective in very preterm children with attention problems and vice versa, and examining intervention effects in both populations instead of in one population and then generalizing the results to the other population, is extremely important.

An important prerequisite for improvement of EF and/or attentional functioning following EF training is plasticity of white matter networks and basal ganglia. In very preterm children, compromised white matter tract integrity and white matter abnormalities are associated with both EF- and attention problems [75–77]. Moreover, damage to the basal ganglia is also associated with attention problems [78] and is frequently observed in children born very preterm [79]. Research has shown that EF training can induce neural changes in brain areas associated with attention in very preterm children [80] and that exercise intervention can induce improvements in white matter integrity in children [81]. It thus seems that the very preterm brain does show the plasticity required for improvement of EF and/or attentional functioning. Possibly, the intensity and duration of the BrainGame Brian training and CWMT in its current forms are not sufficient to induce changes in EF in very preterm children. This is supported by the fact that all children in the EF training condition that had at least one follow-up assessment (24 out of 29, 83%) completed all training sessions, thereby ruling out that not finding any positive results was due to infidelity to the training regimen. Because we did not include brain measures, we cannot rule out that the BrainGame Brian training did induce changes in the brain that did not subsequently lead to changes in attention, behavior or academic performance. However, a recent randomized controlled trial on CWMT in extremely preterm children concluded that brain changes (both structural and functional) generally did not differ between CWMT and placebo conditions [82]. Thus, to induce changes in brain measures and attention, behavior or academic performance, it is likely that more intense and longer training is required. Furthermore, children’s motivation could play a role in the (absence of) training effects. In our study, we did not measure this. However, in a previous pilot study of our group, it was reported that “the majority of parents were positive about BrainGame Brian, in that it is enjoyable and motivating” [50].

Given the growing evidence of ineffectiveness of EF training, alternative interventions should be investigated. One alternative that might come to mind is targeting lower-order cognitive skills with cognitive training, instead of EF (which are higher-order cognitive skills). However, the placebo training condition may be considered as lower-order cognitive training: the child does not train EFs, but still has to remain alert and pay attention to the task in order to perform it correctly. If lower-order cognitive training would be effective, we would therefore expect to find larger improvements following placebo training than following EF training or waitlist condition, which was not the case. A recent meta-analysis on EF training in very preterm children at preschool-age specifically (3–6 years of age) concluded that EF training does have positive results in this population, especially when it is non-computerized and in a group setting [83]. Therefore, we suggest that focus should be directed towards training programs that target younger children, combine exercise and tasks demanding EFs and take place in group settings.

A limitation of the current study is not meeting the required numbers according to our power calculations. However, differences between groups over time were small and not clinically meaningful. Another limitation is that the current study included children with attention problems, and not necessarily children with EF problems. The rationale was that previous studies have shown that EF impairments play an important underlying role in the attention problems observed in very preterm children [28–30]. Last, despite substantial efforts, a large number of teacher questionnaires was missing, especially at the second follow-up visit. However, additional analyses revealed that there were no significant differences in our teacher primary outcome measure at baseline between children with and without the teacher primary outcome measure at second follow-up visit, nor were there any significant differences in demographics between these children. Strengths of the present study are incorporation of both a placebo- and a waitlist control condition, intention-to-treat analyses, comprehensive assessment of a broad range of outcomes using multi-dimensional techniques (questionnaires and performance-based tests) and informants (parent and teacher questionnaires), and assessment of both direct effects and effects five months after the intervention ended.

## Conclusion

Game-formatted EF training is not effective in improving attention, EF or academic functioning in very preterm children with attention problems. Future research may investigate whether alterations to EF training would make such training effective in very preterm children. For example, effectiveness may be enhanced with more ecologically valid and intrinsically rewarding training tasks and/or with longer and more intensive training [84]. Parallel to this, research should also look beyond game-formatted training for interventions to improve EF and associated areas of functioning.

## Electronic supplementary material

Below is the link to the electronic supplementary material.

Supplementary file1 (DOCX 14 kb)
