# Supplementary material for: Executive function training in very preterm children: a randomized controlled trial
Source: Eur Child Adolesc Psychiatry. 2020 May 26;30(5):785–97. doi: 10.1007/s00787-020-01561-0 (PMC7250540; doi:10.1007/s00787-020-01561-0)
Supplement: Supplementary file 1 — Supplementary file1 (DOCX 14 kb) [file 787_2020_1561_MOESM1_ESM.docx]

**Online Resource 1 - Detailed descriptions of measures.

Parent- and teacher rated attention**Parent- and teacher rated attention was measured with the Strengths and Weaknesses of ADHD-symptoms and Normal Behavior (SWAN) questionnaire. This questionnaire contains 18 questions on how well a child performs on behaviors that involve attention, hyperactivity and/or impulsivity compared to peers using a seven-point Likert scale with higher scores indicating poorer attentional skills. Scores on the first nine questions were aggregated into the Inattention subscale, scores on the second nine questions into the Hyperactivity/Impulsivity scale and scores on all questions into a Total Score.

**Parent- and teacher reported daily-life EF**Daily-life EF reported by parents and teachers was measured with the Dutch translation of the Behavior Rating Inventory of Executive Function (BRIEF). This questionnaire consists of 75 questions assessing eight subdomains within EF. Scores of all subdomains were added to compute a Total Score and were subsequently age- and sex-normed to a T-distribution with a mean of 50 and SD of 10, with higher scores indicating more problems in daily-life EF. Furthermore, age- and sex-normed T-scores for the most relevant subdomains (Inhibit, Cognitive Flexibility and Working Memory) were computed as well.

**Verbal working memory**Verbal working memory was measured using the Digit Span Backward subtest of the WISC-III-NL. A sequence of digits was read out loud by the test administrator and children were asked to repeat this sequence in the reverse order. Sequence length started with two and two trials per sequence length were administered until the child was incorrect on both trials for a sequence length. Outcome measure was the product of the largest span length performed correctly at least once, and the total number of correctly repeated sequences.

**Visuospatial working memory**Visuospatial working memory was measured using the Grid Task, backwards condition. On an iPad, a sequence of yellow dots was presented in a 4x4 grid and children were asked to repeat this sequence in the reverse order. Sequence length started with two and per sequence length, four trials (two easy and two difficult) were administered until the child was incorrect on both trials of a difficulty level within a span length. Outcome measure was the product of the largest span length performed correctly at least once, and the total number of correctly repeated sequences.

**Inhibition**Inhibition was measured with the Stop Signal Task. This task consisted of frequent ‘go’ and infrequent ‘stop’ trials and children were instructed to press a button as quickly as possible when a target stimulus was presented, but to refrain from responding when a stop stimulus was presented. Outcome measures were the number of errors on go trials and the Stop Signal Reaction Time (SSRT), which is calculated by subtracting the mean stop signal delay from the mean go reaction time^41^.
 **Cognitive flexibility**Cognitive flexibility was measured with the Multisensory Integration Test (MSIT). In this task, a penguin was presented that tilted to the left or right. In the set condition, children were asked to press the button on the side the penguin tilted to. In the visual shift condition, a visual shift stimulus was presented concurrent with the tilt of the target and children were asked to press the button on the opposite side as the penguin tilted. Outcome measure was Shifting Accuracy loss, defined as the difference in accuracy between the set condition and the visual shift condition, indicating the loss of accuracy in the visual shift condition compared to the set condition, with higher rates corresponding to poorer shifting abilities.
 **Arithmetic**Arithmetic was measured with the TempoTest Automatiseren (TTA). The TTA measures the degree of memorization of mathematical facts. It consists of four pages of 50 arithmetical problems each: one for addition, one for subtraction, one for multiplication and one for division. Children have to complete as many arithmetical problems as possible within two minutes. The outcome measure is total number of arithmetical problems performed correctly. **Technical reading**Technical reading was measured with the Brus Een Minuut Test (B-EMT). This measure consists of one page with four columns of words, 29 each, that are not coherent and differ in length and complexity. Children are asked to read as many words out loud as possible in one minute. The outcome measure is the total number of words read correctly.

**Executive function training in very preterm children: a randomized controlled trial.** *European Child & Adolescent Psychiatry.*
Carolien A. van Houdt^1,2^, MSc, Aleid G. van Wassenaer-Leemhuis, MD, PhD, Jaap Oosterlaan, PhD, Marsh Königs, PhD, Corine Koopman-Esseboom, MD, PhD, A.R. Céleste Laarman, MD, Anton H. van Kaam, MD, PhD, Cornelieke S.H. Aarnoudse-Moens, PhD.
 ^1^ Emma Children’s Hospital, Amsterdam UMC, University of Amsterdam, Neonatology, Meibergdreef 9, Amsterdam, The Netherlands
^2^ Emma Children’s Hospital, Amsterdam UMC, University of Amsterdam, Emma Neuroscience Group, Meibergdreef 9, Amsterdam, The Netherlands
Correspondence to: c.a.vanhoudt@amsterdamumc.nl
